# Diagnostic Performance of the Ovarian-Adnexal Reporting and Data System (O-RADS) Ultrasound Risk Score in Women in the United States

**DOI:** 10.1001/jamanetworkopen.2022.16370

**Published:** 2022-06-09

**Authors:** Priyanka Jha, Akshya Gupta, Timothy M. Baran, Katherine E. Maturen, Krupa Patel-Lippmann, Hanna M. Zafar, Aya Kamaya, Neha Antil, Lisa Barroilhet, Elizabeth A. Sadowski

**Affiliations:** 1 University of California, San Francisco, San Francisco; 2 University of Rochester, Rochester, New York; 3 University of Michigan, Ann Arbor; 4 Vanderbilt University Medical Center, Nashville, Tennessee; 5 University of Pennsylvania, Philadelphia; 6 Stanford University, Stanford, California; 7 University of Wisconsin–Madison, Madison

## Abstract

**Importance:**

The American College of Radiology (ACR) Ovarian-Adnexal Reporting and Data System (O-RADS) ultrasound (US) risk scoring system has been studied in a selected population of women referred for suspected or known adnexal lesions. This population has a higher frequency of malignant neoplasms than women presenting to radiology departments for pelvic ultrasonography for a variety of indications, potentially impacting the diagnostic performance of the risk scoring system.

**Objective:**

To evaluate the risk of malignant neoplasm and diagnostic performance of O-RADS US risk scoring system in a multi-institutional, nonselected cohort.

**Design, Setting, and Participants:**

This multi-institutional cohort study included a population of nonselected women in the United States who presented to radiology departments for routine pelvic ultrasonography between 2011 and 2014, with pathology confirmation imaging follow up or 2 years of clinical follow up.

**Exposure:**

Analysis of 1014 adnexal lesions using the O-RADS US risk stratification system.

**Main Outcomes and Measures:**

Frequency of ovarian cancer and diagnostic performance of the O-RADS US risk stratification system.

**Results:**

This study included 913 women with 1014 adnexal lesions. The mean (SD) age of the patients was 42.4 (13.9 years), and 674 of 913 (73.8%) were premenopausal. The overall frequency of malignant neoplasm was 8.4% (85 of 1014 adnexal lesions). The frequency of malignant neoplasm for O-RADS US 2 was 0.5% (3 of 657 lesions; <1% expected); O-RADS US 3, 4.5% (5 of 112 lesions; <10% expected); O-RADS US 4, 11.6% (18 of 155; 10%-50% expected); and O-RADS 5, 65.6% (59 of 90 lesions; >50% expected). O-RADS US 4 was the optimum cutoff for diagnosing cancer with sensitivity of 90.6% (95% CI, 82.3%-95.9%), specificity of 81.9% (95% CI, 79.3%-84.3%), positive predictive value of 31.4% (95% CI, 25.7%-37.7%) and negative predictive value of 99.0% (95% CI, 98.0%-99.6%).

**Conclusions and Relevance:**

In this cohort study of a nonselected patient population, the O-RADS US risk stratification system performed within the expected range as published by the ACR O-RADS US committee. The frequency of malignant neoplasm was at the lower end of the published range, partially because of the lower prevalence of cancer in a nonselected population. However, a high negative predictive value was maintained, and when a lesion can be classified as an O-RADS US 2, the risk of cancer is low, which is reassuring for both clinician and patient.

## Key Points

**Question:**

What is the diagnostic performance of the Ovarian-Adnexal Reporting and Data System (O-RADS) ultrasound (US) Risk Score in nonselected female patients presenting to radiology departments in United States?

**Findings:**

In this cohort study of 913 patients with 1014 adnexal lesions, an O-RADS US 4 was the optimum cutoff for diagnosing cancer, with a sensitivity of 90.6%, specificity of 81.9%, positive predictive value of 31.4%, and negative predictive value of 99.0%, which was statistically significant.

**Meaning:**

These findings suggest that O-RADS US risk scoring performs within the expected range with a high sensitivity and negative predictive value in nonselected female patients receiving ultrasonography.

## Teaser

This cohort study assesses the frequency of ovarian cancer and evaluates the diagnostic performance of the American College of Radiology Ovarian-Adnexal Reporting and Data System ultrasound risk score among 913 women with suspected or known adnexal lesions.

## Introduction

**Figure 1.**
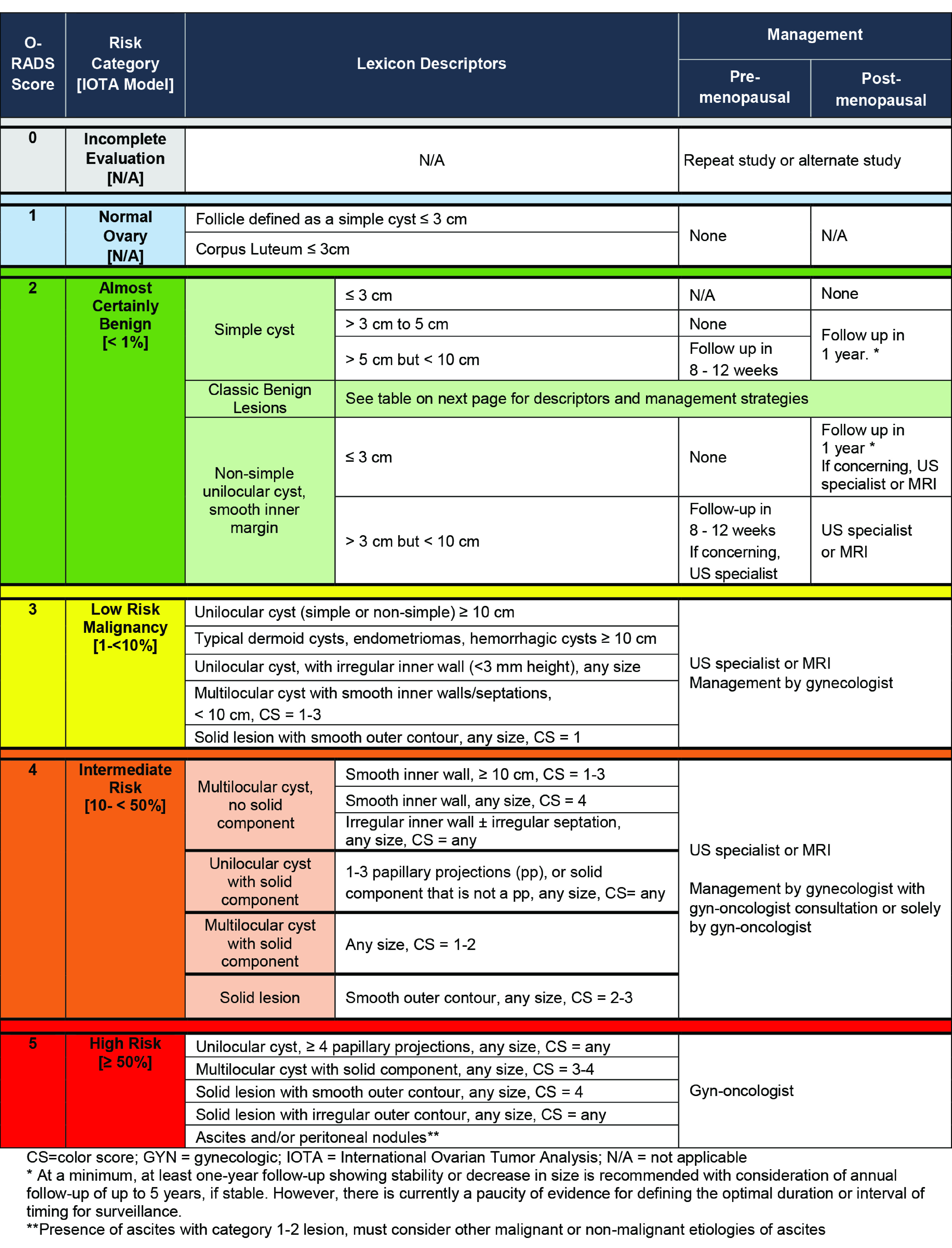
American College of Radiology (ACR) Ovarian-Adnexal Reporting and Data System (O-RADS) Ultrasound Risk Stratification Table The table was reproduced with permission from the American College of Radiology and shows the various risk stratification scores along with respective lesion morphologic descriptors using the O-RADS lexicon and methodology.

When an adnexal lesion is discovered on pelvic ultrasonography, the goal of imaging is to assess if the lesion is benign or potentially malignant. Most adnexal lesions found on ultrasonography imaging are benign and can be classified as simple cysts, hemorrhagic cysts, endometriomas, and dermoids. The risk of malignancy in these lesions is extremely low.^1,2,3,4,5,6,7,8,9^ Lesions not meeting the criteria for 1 of these 4 classic benign lesions carry an increased risk of cancer.^6,7,10,11,12,13,14,15,16^ Multiple ultrasonography-based systems have been developed that use imaging features, such as solid tissue and vascularity, to risk stratify these lesions.^1,3,5,13,17,18^ Most recently, the Ovarian-Adnexal Reporting and Data Systems (O-RADS) Committee was formed under the American College of Radiology (ACR), and this international multidisciplinary group of experts developed the O-RADS ultrasound (US) Risk Stratification and Management System. This system is based on the published lexicon of the O-RADS US working group and from data collected in the International Ovarian Tumour Analysis (IOTA) study phase 1 to phase 3 prospective studies and other studies differentiating benign from potentially malignant lesions.^1,2^ The estimates for malignant neoplasm in the O-RADS US Risk Score table are based on the calculated frequency of malignant neoplasm from women undergoing surgery for known adnexal lesions in the IOTA study (Figure 1). The ACR O-RADS US committee acknowledges the limitations of applying a risk score stratification system founded on a database that includes only surgically resected lesions, which may overestimate the risk of malignant neoplasm in other populations.^2^

Recent studies assessing the O-RADS US risk score have been performed in Europe, Asia, and the Middle East. These retrospective studies have shown that the O-RADS US risk score performs well; however, similar to the IOTA database, these include a high proportion (ie, 80%-100%) of women undergoing surgery for a known adnexal lesion. The frequency of malignant neoplasm in these cohorts approaches 30%.^19,20^ Furthermore, highly experienced radiologists or gynecologists performed the ultrasonography examinations in these previously published studies.^8,18,19,20^ Both the population demographics and the ultrsonography performance are not reflective of clinical practice patterns of many radiology departments in the United States, where women presenting for ultrasonography examinations may not have a known adnexal lesion. When an adnexal lesion is detected, the majority will have benign features, and only lesions with suspicious features will be referred to surgery. As such, the frequency of ovarian cancer in the population of women presenting to radiology is much lower than in a cohort of women being referred to surgery.^14,18^ Lastly, based on prevalent practice patterns in the United States, many of the ultrasonography examinations are performed by sonographers, with the radiologists not scanning the patient following the sonographer’s evaluation. Therefore, this study was undertaken in centers where the technologists scanned the patient. The study patient cohort included patients undergoing surgery and patient with clinical and imaging follow-up, attempting to eliminate the classic verification bias of studies with a high percentage of surgically resected lesions. This study aims to analyze the diagnostic performance of the ACR O-RADS US stratification system in a nonselected population of women in the United States who presented to radiology departments.

## Methods

This cohort study received institutional review board approval from the 6 participating academic institutions and followed the Strengthening the Reporting of Observational Studies in Epidemiology (STROBE) reporting guideline. Informed consent was waived because of the retrospective nature of study.

### Study Population and Participating Institutions

This Health Insurance Portability and Accountability Act–compliant, retrospective multicenter study included nonselected women who presented to academic radiology departments for pelvic ultrasonography. Indications for the ultrasonography examinations were varied and included pelvic pain, fullness, abnormal uterine bleeding, dysmenorrhea, pelvic abnormality on different imaging modality, and suspected mass, among others. Eight investigators participated in the image review, who were fellowship trained in abdominal imaging (1-20 years postresidency experience), including female pelvic ultrasonography, and regularly interpret pelvic ultrasonography examinations as a part of their practice. Specific certification was not required to participate in this study. Between September 2019 and September 2020, investigators at each site reviewed electronic medical records (EMR) and ultrasonography images of consecutive women who underwent a pelvic ultrasonography examination with Doppler in their respective departments between January 2011 and December 2014. The examinations were performed as standard of care and scanned on a variety of ultrasonography units (GE Logiq 9 or E9 (GE Healthcare), Siemens Acuson S2000 (Siemens Healthineers), or Phillips iU22 (Phillips Healthcare) by the ultrasonography technologist. Transabdominal and transvaginal still images and cine clips were part of the ultrasonography examination. Nine hundred of the 1014 adnexal lesions in this study were included in another study comparing the diagnostic performance of using the classic vs nonclassic classification system, whereas, in this study, we assess the diagnostic performance and interobserver agreement of the O-RADS US risk stratification system.^21^

### Inclusion and Exclusion Criteria

Pelvic ultrasonography imaging examinations were reviewed by radiologists and blinded to outcome. Patients with adnexal lesions were recorded. The following findings were excluded: follicles (defined as simple cysts <3 cm in premenopausal women) and corpus luteal cysts and cystic lesions smaller than 1 cm (in postmenopausal women). Classic corpus luteal cysts and follicles were defined per the O-RADS US criteria.^1^

Each investigator reviewed the EMR for the patients at their site and recorded age, menopausal status, and final pathologic diagnosis. For lesions without pathologic diagnosis, either imaging or clinical follow up was used to determine eligibility. Adequate follow-up was defined as pathologic diagnosis, resolution or decrease in size by 10% on follow up imaging, classic lesion on computerized tomography (CT) or magnetic resonance imaging (MRI) (eg, fat seen on CT or MRI to confirm mature cystic teratomas), stability on imaging for greater than 2 years or documented normal pelvic examination greater than 2 years after initial pelvic ultrasonography.

### Image Review and Data Collection

The image review was performed in 2 steps: reproducibility test cohort review and final study population review. First, a group of patients was procured from four sites. A total of 20 deidentified ultrasonography examinations with a variety of O-RADS US categories were sent to a central DropBox at one of the radiologist’s institutions to create the ‘reproducibility test’ population. These ultrasonography examinations were not included in the final study population review.

The second step was the review of all the included examinations of the final study cohort. At each institution, the investigators reviewed all of the images and cine clips available for each study patient. Image review was performed on a PACS including Syngo Dynamics (Siemens Healthineers), McKesson (McKesson Radiology), AGFA (AGFA Heathcare), or GE Centricity (GE Healthcare). The following lesion characteristics were recorded: greatest diameter, side, presence of solid components, number or type of septations, number or greatest diameter of nodules or papillary excrescences, contour of solid component, presence or absence of Doppler flow in solid components, O-RADS US color score, presence of ascites, or peritoneal implants. Doppler images were used to evaluate blood flow and obtain an O-RADS US color score. The site investigator then classified each lesion into a O-RADS US Risk Score category (Figure 1).

After initial O-RADS US classification, a second reader blinded to the primary reader’s classification applied Excel (Microsoft formulas based on recorded cyst features to cross-check the assignment by the primary reader. Any discordant cystic lesions were reviewed for data input integrity. After re-review of the images and verification of presence or absence of an imaging characteristic, the assigned classification was corrected based on consensus between both the primary and secondary reader.

### Statistical Analysis

Continuous measures are summarized as mean (SD), while categorical outcomes are summarized as a proportion (95% CI). Age and menstrual status are summarized on a per-patient basis (n = 913), while all others are summarized on a per-lesion basis (n = 1014). Categorical variables were compared between groups using a Fisher exact test. Receiver operating characteristic (ROC) analysis was used to determine the optimal O-RADS US score to determine malignant neoplasm with maximization of Youden index used to find this optimal point. Sensitivity, specificity, positive predictive value, and negative predictive value were compared between the current study and previous results in the literature independently using a Fisher exact test. The relationship between patient or lesion characteristics and malignant neoplasm was examined independently for each predictor using univariate logistic regression.

Because the data set includes 1014 lesions across 913 patients, there is potential concern for bilateral lesions within a single patient not providing independent data. Additional analysis was therefore performed to account for this by examining only the earliest chronological lesion for each patient. The above analyses were then repeated for 913 lesions across 913 patients. These results are reported in the eTables 1 to 3 in the Supplement.

Interreader agreement for interpretations of the test population was calculated using Fleiss multirater κ, with *P* values comparing with a value of 0. For the resultant κ values, 0 to 0.2 was defined as slight agreement, 0.21 to 0.4 as fair agreement, 0.41 to 0.6 as moderate agreement, 0.61 to 0.8 as substantial agreement, and 0.81 to 1 as almost perfect agreement.^22^ Statistical significance was set at *P* <.05All analyses were performed using MATLAB version R2019b (The Mathworks) and SPSS version 26 (International Business Machines).

## Results

### Patients

**Figure 2.**
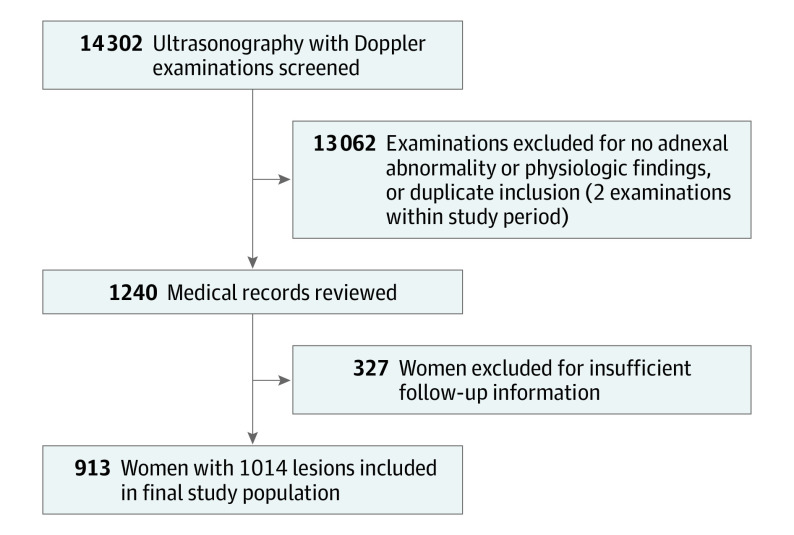
Flowchart of Patient Collection and Inclusion in the Final Study Population

This study included 913 women with 1014 adnexal lesions (Figure 2). The mean (SD) age of the patients was 42.4 (13.9) years, 674 of 913 patients (73.8%) were premenopausal, and 239 of 913 patients (26.2%) were postmenopausal. The rate of cancer was higher in postmenopausal women at 13.8% (33 of 239), compared with 5.2% (35 of 674) in premenopausal women (*P* < .001).

### Follow-up Imaging, Clinical Examination, or Final Pathology

In the final study population, 526 of 1014 lesions (51.9%) were included based on follow-up imaging, 429 of 1014 lesions (42.3%) based on surgical pathology, and 59 of 1014 lesions (5.8%) based on clinical follow-up (Table 1). In our cohort, 929 of 1014 lesions (91.6%) were benign, and 85 of 1014 lesions (8.4%) were malignant. Of the patients who underwent surgery for their adnexal masses, 85 of 429 (19.8%) were found to be malignant. Malignant histopathology encountered ranged from: borderline serous tumors, borderline tumors with papillary features, low grade mucinous tumors, serous tumors of low malignant potential, high grade serous carcinoma, endometrial adenocarcinoma, granulosa cell cancer, dysgerminoma, ovarian carcinosarcoma, stromal carcinoid, and metastatic disease.

**Table 1.** Patient and Lesion Characteristics for the Cohort (Age and Menstrual Status Per Patient [N = 913], Remaining Variables Per Lesion [N = 1014])

Variable	No./Total No. (%)		Odds ratio (95% CI)^a^	*P* value^a^
	Summary of all patients	Frequency of malignant neoplasm		
Age of patients, mean (SD)	42.4 (13.9)	NA	1.03 (1.01-1.05)	<.001
Menstrual status				
Premenopausal	674/913 (73.8)	35/674 (5.2)	NA	NA
Postmenopausal	239/913 (26.2)	33/239 (13.8)	2.78 (1.77-4.37)	<.001
Inclusion based on follow-up				
Surgical pathologic	429/1014 (42.3)	85/429 (19.8)	NA	NA
Imaging follow-up	526/1014 (51.9)	0/526 (0)	NA	NA
Clinical follow-up	59/1014 (5.8)	0/59 (0)	NA	NA
Lesion types				
Simple cyst	306/1014 (30.2)	3/306 (1.0)	NA	NA
Classic hemorrhagic cyst	259/1014 (25.5)	0/259 (0)	0 (0-∞)	>.99^c^
Classic endometrioma	64/1014 (6.3)	1/64 (1.6)	1.60 (0.16-15.66)	.68^c^
Classic dermoid	47/1014 (4.6)	0/47 (0)	0 (0-∞)	>.99^c^
Multilocular cyst without solid tissue^b^	93/1014 (9.2)	1/93 (1.1)	1.10 (0.11-10.68)	.94^c^
Multilocular cyst with solid tissue^b^	108/1014 (10.7)	19/108 (17.6)	21.56 (6.24-74.54)	<.001^c^
Unilocular cyst with solid tissue^b^	83/1014 (8.2)	24/83 (28.9)	41.08 (11.98-140.87)	<.001^c^
Solid	54/1014 (5.3)	37/54 (68.5)	219.82 (61.49-785.88)	<.001^c^
O-RADS US score				
2	657/1014 (64.8)	3/657 (0.5)	NA	NA
3	112/1014 (11)	5/112 (4.5)	10.19 (2.40-43.25)	.002^d^
4	155/1014 (15.3)	18/155 (11.6)	28.64 (8.32-98.59)	<.001^d^
5	90/1014 (8.9)	59/90 (65.6)	414.90 (123.15-1397.90)	<.001^d^
Final outcome				
Benign	929/1014 (91.6)	NA	NA	NA
Malignant	85/1014 (8.4)	NA	NA	NA

Abbreviations: NA, not applicable; O-RADS, Ovarian-Adnexal Reporting and Data System; US, ultrasound.

^a^ Univariate odds ratios (95% CI, *P* value) are the results of univariate logistic regression with malignant neoplasm as the outcome. Reference values for variables with multiple categories are indicated with superscripts.

^b^ Solid tissue: papillary projection, nodule or irregular wall or septation.

^c^ Comparison to simple cyst.

^d^ Comparison to O-RADS US 2.

### Interreader Agreement

Reproducibility test cohort review showed Fleiss κwas 0.81 for assessment of lesion type, 0.90 for the presence of solid components, 0.90 for the presence of vascularity, 0.70 for assessment of presence of septations, 0.68 for the number of septations, 0.71 for the number and contour of solid components, and 0.74 for the assessment of color score. There was only moderate interreader agreement for the type of septation (Fleiss κ= 0.56).

### O-RADS US Category and Lesion Characteristics

Of the 1014 lesions, 657 (64.8%) were classified as O-RADS US 2 (Table 1). O-RADS US 3, 4, and 5 included 112 (11.0%), 155 (15.3%), and 90 (8.9%) of the lesions, respectively (eFigure in the Supplement). Frequency of lesion type distribution in each of the subcategories of O-RADS risk stratification scores 2 to 5 are presented in Table 2.

**Table 2.** Frequency of Malignant Neoplasm in O-RADS US Risk Score 2, 3, 4, and 5 Category and Subcategories

Risk category	No. of lesions	No./No. (%)		
		Frequency of nonneoplastic lesions	Frequency of benign neoplasms	Frequency of malignant neoplasms
O-RADS US: Score 2	657	585/657 (89.0)	69/657 (10.5)	3/657 (0.5)
Simple cyst	294	252/294 (85.7)	40/294 (13.6)	2/294 (0.7)
Hemorrhagic cyst	258	256/258 (99.2)	2/258 (0.8)	0/258 (0)
Endometrioma	62	59/62 (95.2)	2/62 (3.2)	1/62 (1.6)
Dermoid	43	18/43 (41.9)	25/43 (58.1)	0/43 (0)
O-RADS US: Score 3	112	72/112 (64.3)	35/112 (31.3)	5/112 (4.5)
Unilocular cyst or classic lesion >10 cm	19	7/19 (36.8)	11/19 (57.9)	1/19 (5.3)
Unilocular cyst with irregular wall	9	4/9 (44.4)	2/9 (22.2)	3/9 (33.3)
Multilocular cyst <10 cm, smooth inner wall, CS 1-3	78	57/78 (73.1)	20/78 (25.6)	1/78 (1.3)
Solid smooth, any size, CS = 1	6	4/6 (66.7)	2/6 (33.3)	0/6 (0.0)
O-RADS US: Score 4	155	74/155 (47.7)	63/155 (40.6)	18/155 (11.6)
Multilocular cyst ≥10 cm, or any size with smooth inner wall, CS = 4	12	3/12 (25.0)	9/12 (75.0)	0/12 (0.0)
Multilocular cyst >10 cm, any size with irregular wall/septae and any CS	33	21/33 (63.6)	12/33 (36.4)	0/33 (0.0)
Unilocular cyst with solid component, 0-3 papillary projections, any CS	58	26/58 (44.8)	22/58 (39.7)	10/58 (17.2)
Multilocular cyst with solid component, CS = 1-2	50	23/50 (46.0)	19/50 (38.0)	8/50 (16.0)
Solid smooth, any size CS = 2-3	2	1/2 (50.0)	1/2 (50.0)	0/2 (0)
O-RADS US: Score 5	90	19/90 (21.1)	12/90 (13.3)	59/90 (65.6)
Unilocular cyst, ≥4 papillary projections, any CS	11	3/11 (27.3)	1/11 (9.1)	7/11 (63.6)
Multilocular cyst with solid component, CS = 3-4	18	6/18 (33.3)	3/18 (16.7)	9/18 (50.0)
Solid smooth, CS = 4	5	1/5 (20.0)	1/5 (20.0)	3/5 (60.0)
Solid irregular, any CS	15	3/15 (20.0)	1/15 (6.7)	11/15 (73.3)
Lesions scored O-RADS US 3, 4, or 5, plus ascites or peritoneal implants	41	6/41 (14.6)	6/41 (14.6)	29/41 (70.7)

Abbreviations: CS, color score; O-RADS, Ovarian-Adnexal Reporting and Data System; US, ultrasound.

### Frequency of Malignant Neoplasm per O-RADS US Category and Subcategories

The overall frequency of malignant neoplasm in the O-RADS US 2 category was 3 of 657 (0.5%). Three adnexal lesions with an O-RADS US 2 were ultimately diagnosed as malignant; 2 were initially characterized as simple cysts, and 1 as endometrioma. One of the simple cysts was in a premenopausal woman, measuring 4.5 cm on initial examination, that enlarged on follow-up, and was a serous borderline tumor on pathology. The second was a 3.6 cm simple cyst in a postmenopausal woman, that enlarged and developed a small mural nodule on follow-up and was an endometrioid cancer on pathology. Third lesion was a 3.7 cm endometrioma in a premenopausal woman, which enlarged on follow-up and was an endometrioid cancer on pathology.

In the O-RADS US 3 category, the overall frequency of malignant neoplasm was 5 of 112 lesions (4.5%). The subcategory of unilocular cysts with irregular walls had the highest frequency of malignant neoplasm at 3 of 9 lesions (33%); however, the overall number of such lesions was low in our cohort. The most frequently encountered lesion with an O-RADS US 3 was a multilocular cyst without a solid component and smooth inner wall, and lesions in this subcategory were overwhelmingly benign with 1 of 78 lesions (1.3%) risk of malignant neoplasm.

In the O-RADS US 4 category, the overall frequency of malignant neoplasm was 18 of 155 (11.6%). Among lesions with an O-RADS US 4 , unilocularcyst with solid component, 0 to 3 papillary projections, and any color score (CS) were most likely to be malignant (10 of 58 [17.2%]), followed by multilocular cyst with solid components, CS 1 to 2 (8 of 50 [16.0%]). In this cohort, multilocular cysts larger than 10 cm that were (1) any size with smooth inner wall and a CS of 4 (0 of 12 lesions) or (2) any size with irregular walls or septaes and any CS were never found to be malignant (0 of 33 lesions).

In the O-RADS 5 category, the overall frequency of malignant neoplasm was 59 of 90 (65.6%). Solid lesions with irregular outer contour were found to have the highest frequency of cancer at 27 of 33 (81.9%), followed by solid lesions with smooth outer contour at 7 of 9 (77.8%). The lowest rate of cancer (9 of 19 [47.4%]) was in the multilocular cyst with a solid component subcategory.

### Sensitivity, Specificity, PPV, and NPV

The optimal O-RADS US cutoff score of 4 or higher for malignant neoplasm was determined using ROC analysis, with an overall area under the curve (AUC) of 0.92 (95% CI, 0.89-0.95; *P* < .001). This resulted in sensitivity of 90.6% (95% CI, 82.3%-95.9%), specificity of 81.9% (95% CI, 79.3%-84.3%), PPV of 31.4% (95% CI, 25.7%-37.7%), and NPV of 99.0% (95% CI, 98.0%-99.6%). The frequency of malignant neoplasm was significantly increased for lesions with an O-RADS score of 4 or higher, compared with those with an O-RADS score of less than 4 (*P* < .001). Table 3 compares diagnostic performance of the O-RADS US risk score between the current study in a nonselected patient population to previously published studies in populations of women with known adnexal lesion, the majority of whom underwent surgery.^19,20^ The comparison revealed significant differences in specificity and PPV between our study and others already published, with the sensitivities and NPVs not significantly different.

**Table 3.** Comparing Diagnostic Performance Between the Current Study and Previously Published Studies Using the O-RADS US Risk Score ROC-Analysis Using O-RADS 4 and 5 as Malignant Categories

Study	Frequency of malignant lesions, No./No. (%)	TP	FP	FN	TN	Sensitivity (95% CI)	Specificity (95% CI)	PPV (95% CI)	NPV (95% CI)
Current study	85/1014 (8.4)	77	168	8	761	90.6 (82.3-95.9)	81.9 (79.3-84.3)	31.4 (25.7-37.7)	99.0 (98.0-99.6)
Basha et al^19^ (2020)	178/647 (27.5)	172	34	6	435	96.6 (92.8-98.8)	92.8 (90.0-94.9)	83.5 (77.7-88.3)	98.6 (97.1-99.5)
*P* values comparing to current study						.07	<.001	<.001	.59
Cao et al^20 ^(2021)	304/1054 (28.8)	300	126	4	624	98.7 (96.7-99.6)	83.2 (80.3-85.8)	70.4 (65.8-74.7)	99.3 (98.4-99.8)
*P* values comparing to current study						.001	.52	<.001	.56

Abbreviations: NPV, negative predictive value; O-RADS, Ovarian-Adnexal Reporting and Data System; PPV, positive predictive value; ROC, receiver operating characteristic; US, ultrasound.

## Discussion

When an adnexal lesion is discovered on pelvic ultrasonography examination, the goal is to stratify the lesion as benign vs potentially malignant to aid in appropriate follow up, including potential referral to gynecology oncology. Recently, the ACR O-RADS Committee published the O-RADS US Risk Stratification and Management System to help imagers categorize and recommend management for women with adnexal lesions.^1,2^ The O-RADS US risk stratification system is based on an analysis of IOTA data collected between 1999 to 2012 in a population of women referred for surgery of known adnexal lesions.^1,2^ Our results support the applicability of the O-RADS US risk stratification system in a nonselected population of women in the United States. When applied to women in the United States presenting to a radiology departments for pelvic ultrasonography, the calculated frequency of malignant neoplasm for each of the O-RADS US score fell within the lower end of the expected range of the published O-RADS risk stratification algorithm: O-RADS US 2 was 0.5%, (<1%; expected), O-RADS US 3 was 4.5% (<10%; expected), O-RADS US 4 was 11.6% (10%-50%; expected) and O-RADS US 5 was 65.6% (>50% ;expected).^2^ O-RADS US 4 was the optimum cutoff for the diagnosis of cancer, with a sensitivity of 90.6%, specificity of 81.9%, PPV of 31.4%, and NPV of 99.0%.

Two recent retrospective studies^19,20^ evaluated the application of the O-RADS US risk stratification system on large populations of women from the Middle East and Asia. In these studies, nearly all patients underwent surgery for their adnexal lesions: 100% (1054 of 1054) of lesions in the Cao et al^19^ and 77% (499 of 647) lesions in the Basha et al.^20^ This is in contrast to our study, where nonselected patients presented for pelvic ultrasonography for a variety of reasons and only 40% of the lesions were surgically resected. Despite differences in patient cohorts and study design, all 3 studies demonstrated a calculated frequency of malignant neoplasm for each O-RADS US category within the range published by the ACR O-RADS US committee. The ROC-analysis using O-RADS US 4 and 5 as malignant categories demonstrated a sensitivity and specificity of 96.6% and 92.8% in Basha et al^19^ and 98.7% and 83.2% in Cao et al.^20^ These results are similar to our cohort, where the calculated sensitivity and specificity was 90.6% and 81.9%. The trends in sensitivity follow the proportion of surgically resected lesions, being the highest for Cao et al^20^ where all the lesions were surgically resected. The specificity is lowest in our study, where there is a lower prevalence of cancer of 8% vs approximately 30% in the other studies.^19,20^ The inclusion of a high proportion of benign lesions and lower prevalence of ovarian cancer, which would be seen in routine clinical radiology practice, decreases specificity and PPV of ultrasonography for the detection of malignant neoplasm.^6,7,10^ As noted in the guidelines from the ACR O-RADS Committee, the US risk score was designed to maximize the sensitivity for a low prevalence disease process, and it is expected that the specificity and PPV of the O-RADS US system will be lower in populations with a lower prevalence of cancer. In our study of nonselected women, the O-RADS US score performed well in identifying benign lesions (O-RADS US 2 and 3) with a high NPV of 99.0% and showed high sensitivity of 90.6% for the detection of cancer, with the expected decrease in specificity and PPV.

### Limitations

This study had limitations, which include its retrospective design and lack of real-time imaging evaluation. However, this closely mimics routine clinical practice in the United States, where radiologists review the imaging after the patient has left the department. This method also allowed us to have a sufficient duration of follow-up for lesions that were managed nonsurgically. The lesions were consecutive to avoid bias and were not enriched to include an equal proportion of benign and malignant pathologies. This is also reflective of clinical practice where most adnexal lesions are benign. Additionally, the authors acknowledge that imaging acquisition at centers of excellence and interpretation by expert radiologists in this study somewhat limits generalized applicability to other settings. The O-RADS US lexicon and risk stratification system has been designed for use by a general radiologist, and data are needed to validate its use in the nonacademic practice setting.

## Conclusions

The findings of this study suggest that the O-RADS US risk stratification system is a scoring algorithm that can be applied to a nonselected population of women undergoing ultrasonography examinations in radiology departments with similar diagnostic performance compared with previously studied populations. High sensitivity and NPV were maintained to detect ovarian cancer in this population with a lower prevalence of cancer; however, the specificities and PPVs for malignant neoplasm were at the lower end of the expected range published in the O-RADS US Risk score table. The optimal O-RADS US cutoff score for malignant neoplasm was an O-RADS US score of 4 or higher, and gynecology oncology referral in these patients would be advantageous for patient outcomes. Adnexal lesions scored as O-RADS US 2 or 3 had a very low chance of cancer, supporting conservative management for these lesions.

## Notes

Supplement. **eTable 1. **Patient and Lesion Characteristics for the Cohort **eTable 2. **Frequency of Malignant Neoplasm in O-RADS US Risk Score 2, 3, 4, and 5 Category and Subcategories, With Only the Earliest Chronological Lesion Included for Each Patient **eTable 3. **Comparing Diagnostic Performance Between the Current Study and Previously Published Studies Using the O-RADS US Risk Score ROC-Analysis Using O-RADS 4 and 5 as Malignant Categories, With Only the Earliest Chronological Lesion Included for Each Patient in the Current Study **eFigure. **Percentage Distribution of Benign and Malignant Lesions for Ovarian-Adnexal Reporting and Data System (O-RADS) Risk Stratification Scores 2 Through 5
