# Supplementary material for: Diagnostic Performance of the Ovarian-Adnexal Reporting and Data System (O-RADS) Ultrasound Risk Score in Women in the United States
Source: JAMA Netw Open. 2022 Jun 9;5(6):e2216370. doi: 10.1001/jamanetworkopen.2022.16370 (PMC9185186; doi:10.1001/jamanetworkopen.2022.16370)
Supplement: Supplement. — eTable 1. Patient and Lesion Characteristics for the Cohort eTable 2. Frequency of Malignant Neoplasm in O-RADS US Risk Score 2, 3, 4, and 5 Category and Subcategories, With Only the Earliest Chronological Lesion Included for Each Patient eTable 3. Comparing Diagnostic Performance Between the Current Study and Previously Published Studies Using the O-RADS US Risk Score ROC-Analysis Using O-RADS 4 and 5 as Malignant Categories, With Only the Earliest Chronological Lesion Included for Each Patient in the Current Study eFigure. Percentage Distribution of Benign and Malignant Lesions for Ovarian-Adnexal Reporting and Data System (O-RADS) Risk Stratification Scores 2 Through 5 [file jamanetwopen-e2216370-s001.pdf]

Supplemental Online Content

Jha P, Gupta A, Baran TM, et al. Diagnostic performance of the Ovarian-Adnexal Reporting and Data System (O-RADS) ultrasound risk score in women in the United States. *JAMA Netw Open*. 2022;5(6):e2216370. doi:10.1001/jamanetworkopen.2022.16370

eTable 1. Patient and Lesion Characteristics for the Cohort

eTable 2. Frequency of Malignant Neoplasm in O-RADS US Risk Score 2, 3, 4, and 5 Category and Subcategories, With Only the Earliest Chronological Lesion Included for Each Patient

eTable 3. Comparing Diagnostic Performance Between the Current Study and Previously Published Studies Using the O-RADS US Risk Score ROC-Analysis Using O-RADS 4 and 5 as Malignant Categories, With Only the Earliest Chronological Lesion Included for Each Patient in the Current Study

eFigure. Percentage Distribution of Benign and Malignant Lesions for Ovarian-Adnexal Reporting and Data System (O-RADS) Risk Stratification Scores 2 Through 5

This supplemental material has been provided by the authors to give readers additional information about their work.

© 2022 Jha P et al. *JAMA Network Open.*

eTable 1. Patient and Lesion Characteristics for the Cohort[a]

Variable	Category	Summary of all subjects	Frequency of malignancy	Odds Ratio
Age of patients		42.4±13.9	n/a	1.03 (1.01-1.05, p<0.001)
Menstrual Status	Pre-menopausal	73.8% [674/913]	5.2% [35/674]	-
	Post-menopausal	26.2% [239/913]	13.8% [33/239]	2.78 (1.77-4.37, p<0.001)
Inclusion Based On Follow Up	Surgical pathologic	40.3% [368/913]	17.9% [66/368]	
	Imaging follow-up	53.7% [490/913]	0% [0/490]	
	Clinical follow-up	6.0% [55/913]	0% [0/55]	
Lesion Types	Simple Cyst	29.9% [273/913]	0.7% [2/273]	-
	Classic hemorrhagic cyst	26.3% [240/913]	0% [0/240]	0 (0-Inf, p>0.99)[1]
	Classic endometrioma	5.9% [54/913]	1.9% [1/54]	2.6 (0.23-28.7, p=0.45) [1]
	Classic dermoid	4.8% [44/913]	0% [0/44]	0 (0-Inf, p>0.99) [1]
	Multilocular cyst without solid tissue*	12.8% [117/913]	0% [0/117]	0 (0-Inf, p>0.99) [1]
	Multilocular cyst with solid tissue*	7.3% [67/913]	25.4% [17/67]	46.1 (10.3-206, p<0.001)[1]
	Unilocular cyst	8.3% [76/913]	26.3% [20/76]	48.4 (11-213, p<0.001)[1]
	Mostly solid	4.6% [42/913]	61.9% [26/42]	220 (48-1010, p<0.001)[1]
	4 or more solid components	3.2% [29/913]	48.3% [14/29]	114 (38.4-336, p<0.001) [3]
O-RADS US score	2	65% [593/913]	0.5% [3/593]	-
	3	11.1% [101/913]	2.0% [2/101]	4.0 (0.66-24.1, p=0.13)[5]
	4	15.6% [142/913]	10.6% [15/142]	23.2 (6.6-81.4, p<0.001)[5]
	5	8.4% [77/913]	59.7% [46/77]	292 (85.9-991, p<0.001)[5]
Final Outcome	Benign	92.8% [847/913]		-
	Malignant	7.2% [66/913]		-

[a]Patient and lesion characteristics for the cohort, with only the earliest chronological lesion included for subjects with bilateral lesions (n=913). Univariate odds ratios (95% confidence interval, p value) are the results of univariate logistic regression with malignancy as the outcome. Reference values for variables with multiple categories are indicated with superscripts.

*Solid tissue: papillary projection, nodule or irregular wall/septation

[1] Comparison to simple cyst

[2] Comparison to smooth

[3] Comparison to 0 solid components

[4] Comparison to color score 1

[5] Comparison to O-RADS US 2

eTable 2. Frequency of Malignancy in O-RADS US Risk Score 2, 3, 4, and 5 Category and Subcategories, With Only the Earliest Chronological Lesion Included for Each Patient

Risk Category	Number of lesions	Frequency of non-neoplastic lesions	Frequency of benign neoplasms	Frequency of malignant neoplasms
**O-RADS US Score 2**	**593**	**527/593 (88.9%)**	**63/593 (10.6%)**	**3/593 (0.5%)**
Simple cyst	262	85.5% (224/262)	13.7% (36/262)	0.8% (2/262)
Hemorrhagic cyst	239	99.6% (238/239)	0.4% (1/239)	0% (0/239)
Endometrioma	52	94.2% (49/52)	3.8% (2/52)	1.9% (1/52)
Dermoid	40	40% (16/40)	60% (24/40)	0% (0/43)
**O-RADS US Score 3**	**101**	**68.3% (69/101)**	**29.7% (30/101)**	**2.0% (2/101)**
Unilocular cyst or classic lesion >10cm	18	38.9% (7/18)	61.1% (11/18)	0% (0/18)
Unilocular cyst with irregular wall	8	50% (4/8)	25% (2/8)	25% (2/8)
Multilocular cyst <10 cm, smooth inner wall, CS 1-3	70	78.6% (55/70)	21.4% (15/70)	0% (0/70)
Solid smooth, any size, CS = 1	5	60% (3/5)	40% (2/5)	0.0% (0/6)
**O-RADS US Score 4**	**142**	**47.9% (68/142)**	**41.5% (59/142)**	**10.6% (15/142)**
Multilocular cyst ≥10cm, or any size with smooth inner wall, CS = 4	11	18.2% (2/11)	81.8% (9/11)	0.0% (0/12)
Multilocular cyst >10cm, any size with irregular wall/septae and any CS	31	67.7% (21/31)	32.3% (10/31)	0.0% (0/33)
Unilocular cyst with solid component, 0-3 papillary projections, any CS	54	46.3% (25/54)	37.0% (20/54)	16.7% (9/54)
Multilocular cyst with solid component, CS = 1-2	44	43.2% (19/44)	43.2% (19/44)	13.6% (6/44)
Solid smooth, any size CS = 2-3	2	50.0% (1/2)	50.0% (1/2)	0% (0/2)
**O-RADS US Score 5**	**77**	**22.1% (17/77)**	**15.6% (12/77)**	**62.3% (48/77)**
Unilocular cyst, ≥ 4 papillary projections, any CS	9	33.3% (3/11)	11.1% (1/11)	55.6% (5/11)
Multilocular cyst with solid component, CS = 3-4	18	27.8% (5/18)	16.7% (3/18)	55.6% (10/18)
Solid smooth, CS = 4	4	0% (0/4)	25.0% (1/4)	75.0% (3/4)
Solid irregular, any CS	12	25.0% (3/12)	8.3% (1/12)	66.7% (8/12)
Lesions scored O-RADS US 3, 4, or 5, plus ascites or peritoneal implants	34	17.6% (6/34)	17.6% (6/34)	64.7% (22/34)

© 2022 Gha P et al. *JAMA Network Open.*

eTable 3. Comparing Diagnostic Performance Between the Current Study and Previously Published Studies Using the O-RADS US Risk Score ROC-Analysis Using O-RADS 4 and 5 as Malignant Categories, With Only the Earliest Chronological Lesion Included for Each Patient in the Current Study

Study	Frequency of malignant lesions	TP	FP	FN	TN	Sensitivity (95% CI)	Specificity (95% CI)	PPV (95% CI)	NPV (95% CI)
**Current study**	7.2% [66/913]	61	158	5	689	92.4 (83.2-97.5)	81.4 (78.6-83.9)	27.9 (22.0-34.3)	99.3 (98.3-99.8)
**Basha et al.**	27.5%[178/647]	172	34	6	435	96.6 (92.8-98.8)	92.8 (90.0-94.9)	83.5 (77.7-88.3)	98.6 (97.1-99.5)
P values comparing to Current Study						0.17	<0.001	<0.001	0.35
**Cao et a.**	28.8%[304/1054]	300	126	4	624	98.7 (96.7-99.6)	83.2 (80.3-85.8)	70.4 (65.8-74.7)	99.3 (98.4-99.8)
P values comparing to Current Study						0.011	0.36	<0.001	>0.99

© 2022 Gha P et al. *JAMA Network Open.*

eFigure. Percentage Distribution of Benign and Malignant Lesions for Ovarian-Adnexal Reporting and Data System (O-RADS) Risk Stratification Scores 2 Through 5

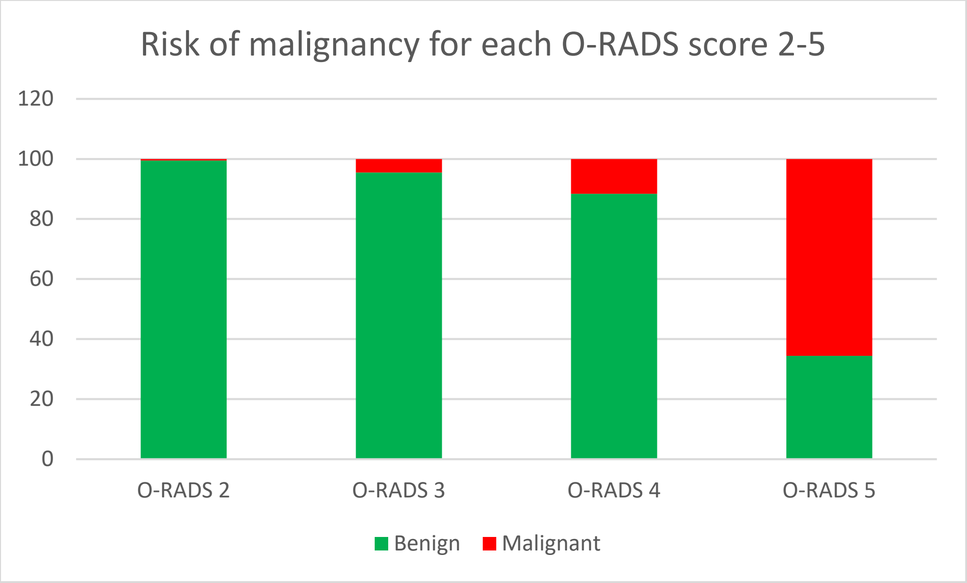

© 2022 Gha P et al. *JAMA Network Open.*
